# On a simple estimation of the proportional odds model under right truncation

**DOI:** 10.1007/s10985-022-09584-2

**Published:** 2023-01-05

**Authors:** Peng Liu, Kwun Chuen Gary Chan, Ying Qing Chen

**Affiliations:** 1 School of Mathematics, Statistics and Actuarial Science, University of Kent, Canterbury, Kent CT2 7FS UK; 2 Department of Biostatistics, University of Washington, Seattle, Washington 98195 USA; 3 Stanford Prevention Research Center, Palo Alto, California 94305 USA

**Keywords:** Biased sampling, Odds ratio, Reverse-time hazard function, 62N02

## Abstract

Retrospective sampling can be useful in epidemiological research for its convenience to explore an etiological association. One particular retrospective sampling is that disease outcomes of the time-to-event type are collected subject to right truncation, along with other covariates of interest. For regression analysis of the right-truncated time-to-event data, the so-called proportional reverse-time hazards model has been proposed, but the interpretation of its regression parameters tends to be cumbersome, which has greatly hampered its application in practice. In this paper, we instead consider the proportional odds model, an appealing alternative to the popular proportional hazards model. Under the proportional odds model, there is an embedded relationship between the reverse-time hazard function and the usual hazard function. Building on this relationship, we provide a simple procedure to estimate the regression parameters in the proportional odds model for the right truncated data. Weighted estimations are also studied.

## Introduction

Truncation is common in survival analysis where the incomplete nature of the observations is due to a systematic biased selection process originated in the study design. Right truncated data arise naturally when an incubation period (i.e., the time between disease incidence and the onset of clinical symptoms) cannot be observed completely in a retrospective study. In survival analysis, right truncation will lead to biased sampling in which shorter observations will be oversampled (Gürler 1996). For example, to study AIDS caused by blood transfusion (Lagakos et al. 1988), the incubation period is the time from a contaminated blood transfusion to the time when symptoms and signs of AIDS are first apparent. However, in those studies, the following-up period are usually limited. Therefore, only those developed AIDS before the end of study can be identified.

Many authors have studied right truncated data: Woodroofe (1985) and Wang et al. (1986) focused on the asymptotic properties the product limit estimator under random truncation. Keiding and Gill (1990) studied asymptotic properties of random left truncation estimator by a reparametrization of the left truncation model as a three-state Markov process. Lagakos et al. (1988) considered nonparametric estimation and inference of right truncated data by treating the process in reverse time, they showed that $\lambda ^{B}(t)=\lambda (\tau -t)$, where $\tau$ is the study duration, $\lambda ^{B}(t)$ and $\lambda (t)$ are reverse-time hazard and forward-time hazard, respectively. The authors also discussed the implications and limitations of introducing reverse time hazard to analyze right truncated data. Gross (1992) further explained the necessity of reverse time hazard in the Cox model setting.

However, in most of the current literature, researchers study right truncated data in nonparametric setting, fairly few studied semiparametric models, among them, Kalbfleisch and Lawless (1989) formulating the Cox model on the reverse time hazard (or retro hazard, Lagakos et al. (1988); Keiding and Gill (1990)). For other related work on reverse time hazard, please refer to Gross (1992); Chen et al. (2004), among others.

In this paper, we study right truncated data under a semiparametric proportional odds model. Different from a proportional hazards model, the reverse-time hazard in proportional odds model has a simple log-linear relationship with the forward-time hazard, which leads to an intuitive estimator. While Sundaram (2009)’s method can also be adapted to proportional odds model for right truncated data, she focused on applying a reversed-time argument to an estimator for left truncated data. Our estimator, on the other hand, utilize a direct relationship between the reverse-time hazard, the forward-time hazard and the baseline odds function, so that we obtain a simpler estimator. Weighted functions are also being inserted into the estimating equation to obtain more efficient estimates.

The rest of the paper is organized as follows. Section 2 describes the inference procedure as well as asymptotic results, Sect.  3 shows simulation and real data results, Sect. 5 provides some discussion. Proof of theorems are left into the Appendix part.

## Inference procedure

Assume that the failure time of interest *T* follows the semiparametric proportional odds model:

1 $$\begin{aligned} \log \left\{ \frac{1-S(t\mid Z)}{S(t\mid Z)}\right\} =\alpha (t)+Z^\top \beta , \end{aligned}$$

and the observed failure time is subject to a right truncation time variable *R*. The observed data is $(T_i,R_i),~i=1,\ldots ,n$, where $T_i\le R_i$. Let $\tau$ be the study duration, which is greater than $\max \{T_1,T_2,\ldots ,T_n\}$. An (observed) reverse-time sample, $(T_i^{*},R_i^{*}),~i=1,\ldots ,n$ can be constructed, where $T^{*}=\tau -T,~R^{*}=\tau -R$, so that $T^{*}$ is left truncated by the variable $R^{*}$. Denote $({\tilde{T}}^{*},{\tilde{R}}^{*})$ as the reverse-time sample (potentially truncated). Then the hazard function of ${\tilde{T}}^{*}$ is a quantity originated in $\tau$ and counts backward in time. The reverse hazard and cumulative reverse hazard function of backward recurrence time is defined as

$$\begin{aligned}{} & {} \lambda ^B(t\mid Z)=\lim _{\Delta t\rightarrow 0}\frac{\text {Pr}\{{\tilde{T}}^{*}\in (t-\Delta t,t]\mid {\tilde{T}}^{*}\le t, Z\}}{\Delta t}=\frac{f(t\mid Z)}{F(t\mid Z)},\\{} & {} \Lambda ^B(t\mid Z)=\int _t^{\tau }\lambda ^B(s\mid Z)ds. \end{aligned}$$

We would like to mention that a similar definition of the reverse hazard can also be found in Kalbfleisch and Lawless (1989) and Jiang (2011). Denote $v(t)=\exp (\alpha (t))$, and $\lambda (t)=f(t)/S(t)$ as the forward-time hazard, then

$$\begin{aligned} \log \lambda (t\mid Z)-\log \lambda ^{B}(t\mid Z)= & {} \alpha (t)+Z^\top \beta , \lambda ^B(t\mid Z)\\ {}= & {} \frac{1}{\{1+v(t)\exp (Z^\top \beta )\}v(t)}\frac{dv(t)}{dt}. \end{aligned}$$

Consider the counting process

$$\begin{aligned} N_i(t)=I(t\le T_i\le R_i), Y_i(t)= I(T_i\le t\le R_i), \end{aligned}$$

and denote

$$\begin{aligned} M_i(t,\beta )=N_i(t)-\int _t^{\tau }Y_i(s)\frac{1}{\{\exp (Z_i^\top \beta )v(s)+1\}v(s)}dv(s). \end{aligned}$$

Then $M_i(t,\beta )$ is a martingale with respect to the self-exciting (canonical) filtration (Keiding and Gill 1990; Stralkowska-Kominiak and Stute 2009) and

2 $$\begin{aligned} M_i(dt,\beta )=dN_i(t)+Y_i(t)\frac{1}{\{\exp (Z_i^\top \beta )v(t)+1\}v(t)}dv(t). \end{aligned}$$

Multiply both sides of (2) by $\{\exp (Z_i^\top \beta )v(t)+1\}$ and summing over *n* observations,

3 $$\begin{aligned}{} & {} \sum _{i=1}^n\{\exp (Z_i^\top \beta )v(t)+1\}dN_i(t)+\sum _{i=1}^n Y_i(t)\frac{dv(t)}{v(t)}\nonumber \\{} & {} \qquad =\sum _{i=1}^n\{\exp (Z_i^\top \beta )v(t)+1\}M_i(dt,\beta ). \end{aligned}$$

Divide both left-hand side and right-hand side by $\sum _{i=1}^nY_i(t)$, we obtain:

$$\begin{aligned} \frac{\sum _{i=1}^n\{\exp (Z_i^\top \beta )v(t)+1\}d N_i(t)}{\sum _{i=1}^n Y_i(t)}+\frac{d v(t)}{v(t)}=\frac{\sum _{i=1}^n\{\exp (Z_i^\top \beta )v(t)+1\}M_i(d t,\beta )}{\sum _{i=1}^n Y_i(t)}. \end{aligned}$$

which is equivalent to:

4 $$\begin{aligned}{} & {} v(t)\frac{\sum _{i=1}^n\exp (Z_i^\top \beta )d N_i(t)}{\sum _{i=1}^n Y_i(t)}+\frac{\sum _{i=1}^n d N_i(t)}{\sum _{i=1}^n Y_i(t)}+\frac{d v(t)}{v(t)}\nonumber \\{} & {} \qquad = \sum _{i=1}^n\frac{\exp (Z_i^\top \beta )v(t)+1}{\sum _{i=1}{n}Y_i(t)}M_i(dt,\beta ). \end{aligned}$$

Denote the left-hand side of (4) as:

$$\begin{aligned} U(\beta ,dt)=\frac{dv(t)}{v(t)}+p_n(t)dt-q_n(t,\beta )v(t)dt, \end{aligned}$$

where

$$\begin{aligned} p_n(t)dt=\frac{\sum _{i=1}^ndN_i(t)}{\sum _{j=1}^nY_j(t)}, q_n(t,\beta )dt=-\frac{\sum _{i=1}^n\exp (Z_i^\top \beta )dN_i(t)}{\sum _{j=1}^nY_j(t)}. \end{aligned}$$

From standard counting process arguments (Anderson and Gill, 1982;Aalen10), we know that the stochastic integral with respect to the counting process martingale $M_i(dt,\beta )$ is also a martingale, motivate by the following equation

$$\begin{aligned} E\left[ \frac{1}{n}U(\beta ,dt)\right] = E\left[ \frac{1}{n}\sum _{i=1}^n\frac{\exp (Z_i^\top \beta )v(t)+1}{\sum _{i=1}{n}Y_i(t)}M_i(dt,\beta ) \right] . \end{aligned}$$

We construct the following estimating equation

5 $$\begin{aligned} \frac{1}{n}U(\beta ,dt)=0. \end{aligned}$$

Only *v*(*t*) is unknown in (5), let the estimate of *v*(*t*) be ${\hat{v}}_n(t,\beta )$. Denote

$$\begin{aligned} P_n(t)=\exp \left\{ \int _t^{\tau }\frac{\sum _{i=1}^ndN_i(s)}{\sum _{j=1}^nY_j(s)} \right\} , Q_n(t,\beta )=\int _t^{\tau }\frac{\sum _{i=1}^n\exp (Z_i^\top \beta )dN_i(s)}{\sum _{j=1}^nY_j(s)}, \end{aligned}$$

then

6 $$\begin{aligned} {\hat{v}}_n(t,\beta ) =\frac{P_n(t)}{\int _t^{\tau }P_n(s)Q_n(ds,\beta )}. \end{aligned}$$

Multiply (2) by $Z_i\{\exp (Z_i^\top \beta )v(t)+1\}/n$ and summing over *n* observations, we obtain

7 $$\begin{aligned}{} & {} \frac{1}{n}\sum _{i=1}^nZ_i\left[ \{\exp (Z_i^\top \beta )v(t)+1\}dN_i(t)+ Y_i(t)\frac{dv(t)}{v(t)}\right] \nonumber \\{} & {} \qquad =\frac{1}{n}\sum _{i=1}^nZ_i\{\exp (Z_i^\top \beta )v(t)+1\}M_i(dt,\beta ). \end{aligned}$$

By virtue of the same idea of (5), take integration on both sides of (7), we can also construct another equation:

8 $$\begin{aligned} \frac{1}{n}\sum _{i=1}^n\int _0^{\tau }Z_i\left[ \{\exp (Z_i^\top \beta )v(t)+1\}dN_i(t)+ Y_i(t)\frac{dv(t)}{v(t)}\right] =0 \end{aligned}$$

Substituting (6) into (8), we can obtain the estimate of $\beta$ by solving the following equation:

$$\begin{aligned} \frac{1}{n}\sum _{i=1}^n\int _0^{\tau }Z_i\left[ \left\{ \exp (Z_i^\top \beta ){\hat{v}}_n(t,\beta )+1 \right\} dN_i(t) +Y_i(t)\frac{{\hat{v}}_n(dt,\beta )}{{\hat{v}}_n(t,\beta )} \right] =0. \end{aligned}$$

Moreover, since

$$\begin{aligned} \frac{{\hat{v}}_n(dt,\beta )}{{\hat{v}}_n(t,\beta )}=-\frac{\sum _{k=1}^ndN_k(t)}{\sum _{l=1}^nY_l(t)} -\frac{\sum _{k=1}^n\exp (Z_k^\top \beta )dN_k(t)}{\sum _{l=1}^nY_l(t)}{\hat{v}}_n(t,\beta ), \end{aligned}$$

then

$$\begin{aligned} \frac{1}{n}\sum _{i=1}^n\int _0^{\tau }\left\{ Z_i-{\bar{Z}}(t)\right\} \left\{ \exp (Z_i^\top \beta ){\hat{v}}_n(t,\beta )+1\right\} dN_i(t)=0, \end{aligned}$$

where

$$\begin{aligned} {\bar{Z}}(t)=\frac{\sum _{i=1}^nZ_iY_i(t)}{\sum _{j=1}^nY_j(t)}. \end{aligned}$$

Finally, let

9 $$\begin{aligned} S_n(\beta )=\frac{1}{n}\sum _{i=1}^n\int _0^{\tau }\left\{ Z_i-{\bar{Z}}(t)\right\} \left\{ \exp (Z_i^\top \beta ){\hat{v}}_n(t,\beta )+1\right\} dN_i(t), \end{aligned}$$

and denote the solution of $S_n(\beta )=0$ be ${\hat{\beta }}_n$, we have the following theorem:

### Theorem 1

Under assumptions A1-A4 in the Appendix, $\sqrt{n}({\hat{\beta }}_n-\beta _0)$ converges weakly to a mean-zero normal distribution, with covariance matrix $U^{-1}V(U^{-1})^\top$, where *V* is the covariance matrix of $\sqrt{n}S_n(\beta _0)$, $U=\lim _{n\rightarrow \infty }\{\partial S_n(\beta )/\partial \beta \}\mid _{\beta =\beta _0}$. The *k*th row of *U* is:

$$\begin{aligned}{} & {} \mathop {\lim }\limits _{n\rightarrow \infty }\frac{1}{n}\sum _{i=1}^n\int _0^{\tau }\left\{ Z_i-{\bar{Z}}(t)\right\} \\{} & {} \quad \times \left\{ Z_{ik}\exp (Z_i^\top \beta _0){\hat{v}}_n(t,\beta _0)+\exp (Z_i^\top \beta _0) \frac{\partial {\hat{v}}_n(t,\beta )}{\partial \beta _k}\mid _{\beta =\beta _0} \right\} dN_i(t). \end{aligned}$$

### Remark

For proportional odds model with the normal logit link:

10 $$\begin{aligned} \log \left\{ \frac{S(t\mid Z)}{1-S(t\mid Z)}\right\} =\alpha (t)+Z^\top \beta . \end{aligned}$$

Define

$$\begin{aligned} {\tilde{M}}_i(t,\beta )=N_i(t)-\int _t^{\tau }Y_i(s) \frac{\exp (Z^\top \beta )}{1+\exp (Z^\top \beta )v(s)} dv(s), \end{aligned}$$

we claim that ${\tilde{M}}_i(t,\beta )$ is a martingale. Recall that $v(t)=\exp (\alpha (t))$, following (10), we have

$$\begin{aligned} S(t|Z)=\frac{\exp (\alpha (t)+Z^\top \beta )}{1+\exp (Z^\top \beta )v(t)}, \end{aligned}$$

as a result, we can obtain

$$\begin{aligned} f(t|Z)=\frac{\exp (Z^\top \beta )v^{\prime }(t)}{(1+\exp (Z^\top \beta )v(t))^2}, F(t|Z)=\frac{1}{1+\exp (Z^\top \beta )v(t)}. \end{aligned}$$

Following the definition of reverse hazard in Sect.  2, we can write the reverse hazard as

$$\begin{aligned} {\tilde{\lambda }}^B(t|Z)=\frac{f(t|Z)}{F(t|Z)}=\frac{\exp (Z^\top \beta )v^{\prime }(t)}{1+\exp (Z^\top \beta )v(t)}. \end{aligned}$$

From the general definition of martingale in Fleming and Harrington (1991) (pp. 25), we can easily show that ${\tilde{M}}_i(t,\beta )$ is a martingale. While for model (1),

$$\begin{aligned} \lambda ^B(t|Z)=\frac{v^{\prime }(t)}{1+\exp (Z^\top \beta )v(t)}, \end{aligned}$$

and $N_i(t)-\int _{t}^{\tau }Y_i(s)\lambda ^B(t|Z)dt$ is the martingale.

The corresponding estimating equation under model (10) has the following form

11 $$\begin{aligned} S_n^{(1)}(\beta )=\sum _{i=1}^n\int _0^{\tau }\left\{ Z_i-{\bar{Z}}(t,\beta )\right\} \left\{ \exp (Z_i^\top \beta ){\hat{v}}_n(t,\beta )+1 \right\} dN_i(t), \end{aligned}$$

where

$$\begin{aligned} {\bar{Z}}(t,\beta )=\frac{\sum _{i=1}^nZ_iY_i(t)\exp (Z_i^\top \beta )}{\sum _{j=1}^nY_j(t)\exp (Z_j^\top \beta )}. \end{aligned}$$

Equation (11) also can be used to estimate $\beta$, however, comparing with (9), (11) is more complicated and more computational intensive, while the derivative of (9) with respect to $\beta$ can be easily obtained. As a result, (9) can be easily solved by the newton raphson algorithm. In the following simulations, we will use estimating equation (9).

In addition to the unweighted object function (9), weighted object function can also being included to obtain a class of weighted estimators of $\beta _0$. This procedure is often used to minimize the sandwich estimate as well as improve the efficiency. The weighted version of object function is

12 $$\begin{aligned}{} & {} S_{n,W}(\beta )\nonumber \\{} & {} \quad =\frac{1}{n}\sum _{i=1}^n\int _0^{\tau }W_n(t)\left\{ Z_i-{\bar{Z}}(t)\right\} \left\{ \exp (Z_i^\top \beta ){\hat{v}}_n(t,\beta )+1\right\} dN_i(t)=0, \end{aligned}$$

here $W_n(t)$ is a predictable weight function with respect to the canonical filtration which converges to a non-random function *w*(*t*). One of the common used weight function is the Prentice-Wilcoxon type function $W_{n1}(t)={\hat{S}}_{LB}(t)$, where ${\hat{S}}_{LB}(\cdot )$ is the Lynden Bell estimate of the baseline survival function for right truncated failure time data. Denote the corresponding estimate of $\beta$ as ${\hat{\beta }}_{n,w}$. Then we have the following theorem:

### Theorem 2

Under the same assumptions as Theorem 1, when $n\rightarrow \infty$, for a prespecified weight function $W_n(\cdot )\rightarrow w(\cdot )$, $\sqrt{n}({\hat{\beta }}_{n,w}-\beta _0)$ converges weakly to a mean-zero normal distribution, with covariance matrix $U_w^{-1}V_w(U_w^{-1})^\top$, where $V_w$ is the covariance matrix of $\sqrt{n}S_{n,w}(\beta _0)$, $U_w=\lim _{n\rightarrow \infty }\{\partial S_{n,w}(\beta )/\partial \beta \}\mid _{\beta =\beta _0}$. The *k*th row of $U_w$ is:

$$\begin{aligned}{} & {} \mathop {\lim }\limits _{n\rightarrow \infty }\frac{1}{n}\sum _{i=1}^n\int _0^{\tau } W_n(t)\left\{ Z_i-{\bar{Z}}(t)\right\} \left[ Z_{ik}\exp (Z_i^\top \beta _0){\hat{v}}_n(t,\beta _0)\right. \\{} & {} \quad \left. +\exp (Z_i^\top \beta _0) \left\{ \partial {\hat{v}}_n(t,\beta )/\partial \beta _k\right\} \mid _{\beta =\beta _0} \right] dN_i(t). \end{aligned}$$

Recently, many people considered problem of finding the optimal weight in a weighted estimating equation, including Chen and Cheng (2005); Chen and Wang (2000); Chen et al. (2012), among others. To achieve this goal, we only need to find the *w*(*t*) such that $U_w(\beta _0)^{-1}V_w(\beta _0)U_w(\beta _0)^{-1}$ achieves the minimum. Since both the empirical weight function $W_n(t)$ and its limit *w*(*t*) do not rely on unknown parameter $\beta _0$, it is reasonable to set $\beta _0=0$. Another explanation for letting $\beta _0=0$ is that it represents the baseline distribution. Therefore, let $\beta _0=0$, then we have:

13 $$\begin{aligned} U_w(\beta _0){} & {} =\mathop {\lim }\limits _{n\rightarrow \infty }\frac{1}{n}\sum _{i=1}^n\int _0^{\tau } W_n(t)\left\{ Z_i-{\bar{Z}}(t)\right\} Z_{i}\exp (Z_i^\top \beta _0)v(t)dN_i(t)\nonumber \\{} & {} =\mathop {\lim }\limits _{n\rightarrow \infty }\frac{1}{n}\sum _{i=1}^n\int _0^{\tau } W_n(t)\left\{ Z_i-{\bar{Z}}(t)\right\} ^{\otimes 2} \exp (Z_i^\top \beta _0)v(t)dN_i(t)\nonumber \\{} & {} =\mathop {\lim }\limits _{n\rightarrow \infty }\frac{1}{n}\sum _{i=1}^n\int _0^{\tau } W_n(t)\left\{ Z_i-{\bar{Z}}(t)\right\} ^{\otimes 2}Y_i(t) \frac{\exp (Z_i^\top \beta _0)v^{\prime }(t)}{\exp (Z_i^\top \beta _0)v(t)+1} dt\nonumber \\{} & {} =\mathop {\lim }\limits _{n\rightarrow \infty }\frac{1}{n}\sum _{i=1}^n\int _0^{\tau } W_n(t)\left\{ Z_i-{\bar{Z}}(t)\right\} ^{\otimes 2}Y_i(t) \frac{v^{\prime }(t)}{v(t)+1} dt. \end{aligned}$$

14 $$\begin{aligned} V_w(\beta _0){} & {} =\mathop {\lim }\limits _{n\rightarrow \infty }\frac{1}{n}\sum _{i=1}^n\int _0^{\tau } W_n(t)^2\left\{ Z_i-{\bar{Z}}(t)\right\} ^{\otimes 2}\nonumber \\{} & {} \quad \times \left\{ \exp (Z_i^\top \beta _0)v(t)+1\right\} ^2Y_i(t)\frac{1}{\left\{ \exp (Z_i^\top \beta _0)v(t)+1\right\} v(t)}v^{\prime }(t)dt\nonumber \\{} & {} =\mathop {\lim }\limits _{n\rightarrow \infty }\frac{1}{n}\sum _{i=1}^n\int _0^{\tau } W_n(t)^2\left\{ Z_i-{\bar{Z}}(t)\right\} ^{\otimes 2}Y_i(t)\left\{ \exp (Z_i^\top \beta _0)v(t)+1\right\} \frac{v^{\prime }(t)}{v(t)}dt\nonumber \\{} & {} =\mathop {\lim }\limits _{n\rightarrow \infty }\frac{1}{n}\sum _{i=1}^n\int _0^{\tau } W_n(t)^2\left\{ Z_i-{\bar{Z}}(t)\right\} ^{\otimes 2}Y_i(t)\left\{ v(t)+1\right\} \frac{v^{\prime }(t)}{v(t)}dt \end{aligned}$$

Apply the Cauchy-Schwarz inequality to $U_w(\beta _0)^{-1}V_w(\beta _0)U_w(\beta _0)^{-1}$ and let $\beta _0=0$, then it follows that the optimal weight is proportional to

15 $$\begin{aligned} w(t)=\frac{v(t)}{(v(t)+1)^2}=S(t)\left\{ 1-S(t)\right\} , \end{aligned}$$

which minimize the variance of ${\hat{\beta }}_n$. Since when (15) holds, we have

$$\begin{aligned}{} & {} U_w(\beta _0)=\mathop {\lim }\limits _{n\rightarrow \infty }\frac{1}{n}\sum _{i=1}^n\int _0^{\tau } \left\{ Z_i-{\bar{Z}}(t)\right\} ^{\otimes 2}Y_i(t) \frac{v(t)v^{\prime }(t)}{(v(t)+1)^3} dt,\\{} & {} V_w(\beta _0)=\mathop {\lim }\limits _{n\rightarrow \infty }\frac{1}{n}\sum _{i=1}^n\int _0^{\tau } \left\{ Z_i-{\bar{Z}}(t)\right\} ^{\otimes 2}Y_i(t)\frac{v(t)v^{\prime }(t)}{(v(t)+1)^3}dt, \end{aligned}$$

which means when $\beta _0=0$, given $w(t)=S(t)\{1-S(t)\}$, we have $U_w(\beta _0)^{-1}V_w(\beta _0)U_w(\beta _0)^{-1}$ achieves the minimum value $U_w(\beta _0)^{-1}$ (or equivalently $V_w(\beta _0)^{-1}$).

In simulation, let $W_{n2}(t) ={\hat{S}}_{LB}(t)\left\{ 1-{\hat{S}}_{LB}(t)\right\}$, the results are shown in Table , it can be seen that the weight $W_{n2}(t)$ achieve the minimal variance among the three estimators.

## Simulation and real data

We perform simulation studies to evaluate the finite sample properties of the proposed estimator. In simulation, let $\alpha (t)=3\log t$, $\beta _0=(1,0.5)^\top$, $Z_1$ is a continuous variable follows a uniform distribution from 0 to 2, $Z_2$ is a discrete variable follows a Bernoulli distribution with probability 0.5. The failure time variable is generated from model (1). The right truncation variable follows a uniform distribution from 0 to 4. This makes the truncation rate equals to 20%. For each simulation, 1000 datasets are generated, in each dataset, there are *n* observations, $n=300,400,500, 600$, respectively. $W_{n1}(t)$ and $W_{n2}(t)$ are chosen as the weight functions in weighted estimating equations. As is shown in Table 1, three estimation equations yield unbiased estimates and the empirical coverage probability is around nominal level 95%, when weighted function is incorporated into the estimation equation, the efficiency is greatly improved, and the variance achieve minimal for $W_{n2}(t)$ under three estimates.

As pointed out by one of the referees and the associate editor, Shen et al. (2017) also studied right truncated data under linear transformation models, and we know that when the error term in the linear transformation model follows logistic distribution (Fine et al., 1998), the model becomes the proportional odds model. Let

$$\begin{aligned}{} & {} N_i^{\dag }(t)=I(\tau -T_i\le t)=I(T_i\ge \tau -t),\\{} & {} Y_i^{\dag }(t)=I(\tau -R_i\le t\le \tau -T_i)=I(T_i\le \tau -t\le R_i), \end{aligned}$$

then the estimating equations (3) and (4) in Shen et al. (2017) can be written as

$$\begin{aligned}{} & {} U(\beta ,\alpha (\tau -t))\\{} & {} \quad =\sum _{i=1}^{n}\int _{-\infty }^{\tau }Z_i\left[ dN_i(t)-Y_i(t)d\left( \log \frac{\exp (Z_i^\top \beta +\alpha (\tau -t))}{1+\exp (Z_i^\top \beta +\alpha (\tau -t))} \right) \right] =0,\\{} & {} \qquad \sum _{i=1}^n\left[ dN_i(t)-Y_i(t)d\left( \log \frac{\exp (Z_i^\top \beta +\alpha (\tau -t))}{1+\exp (Z_i^\top \beta +\alpha (\tau -t))} \right) \right] =0. \end{aligned}$$

We recognize that Shen et al. (2017)’s methodology is general and works for all the linear transformation models, including the proportional odds model. However, our approach will be more convenient compared with Shen et al. (2017)’s under the proportional odds model, since our approach has a simpler form, and the estimation of the intercept $\alpha (t)$ can be done beforehand and plugged in the final estimating equation, while Shen et al. (2017) can not achieve this and their estimation produce involves a complicated iteration which increases the risk of non-convergence. Besides, Shen et al. (2017) only deal with the reverse time but not the reverse hazard function, and we utilize the relationship between the reverse hazard function and the forward-time hazard function and produced a more intuitive estimator.

We conduct simulations for Shen et al. (2017)’s method and the results are reported in Table 1. The code was obtained from the authors via personal communication. However, one of the authors, Prof. Pao-Sheng Shen mentioned that they were unable to calculate the asymptotic variance and coverage probabilities, the existing results in their paper contain some errors, and their current code only consists of bias and standard error. As a result, we only report bias and standard error of Shen et al. (2017)’s method. All the simulations were conducted under the same model as ours. We also want to mention that we found the computation speed is very slow for Shen et al. (2017)’s method, though asymptotic variance and coverage probability were not calculated, their method is still more than 3 times slower than ours under the same model setting and the sample size. The SSE of Shen et al. (2017)’s method is smaller than our unweighted estimator, but is bigger than the two weighted estimators. For the second approach in their paper, i.e. the conditional maximum-likelihood approach, since the bias is large, we did not perform further comparisons here. We would like to mention that the large bias of the conditional maximum-likelihood approach is also confirmed in Vakulenko-Lagun et al. (2020).

As suggested by one of the reviewers, we also perform simulations without accounting for the truncation, and the results are shown in Table . We choose the truncation distribution as uniform distributions from 0 to 4, 2 and 1, respectively, which corresponds to 20% truncation rate (mild truncation), 40% truncation rate (moderate truncation) as well as 70% truncation rate (heavy truncation). As we can see from Table 2, all the estimators are biased, and a larger truncation rate will lead to a bigger bias and variance, though for the same truncation, variances will decrease when the sample sizes increase. These results also coincide with Table 2.1 (pp. 20) in Rennert (2018) and Table 1 in Rennert and Xie (2018), though the two articles deal with the doubly truncated data under the Cox model.

**Table 1:** Simulation results

		${\hat{\beta }}_n^{(1)}$					${\hat{\beta }}_n^{(2)}$			
n		Bias$\times 10^3$	SSE$\times 10^3$	SEE$\times 10^3$	Cov(%)		Bias$\times 10^3$	SSE$\times 10^3$	SEE$\times 10^3$	Cov(%)
300	Unweight	31	329	342	96		8	319	338	97
	Prentice-Wilcoxon	30	249	277	95		18	268	278	95
	$W_{n2}(t)$	23	227	258	93		8	254	261	93
	Shen et al. (2017)	-2	271	NA	NA		39	274	NA	NA
400	Unweight	20	278	289	96		7	271	288	96
	Prentice-Wilcoxon	12	213	240	96		12	227	241	95
	$W_{n2}(t)$	8	194	222	94		5	211	225	94
	Shen et al. (2017)	-9	210	NA	NA		7	251	NA	NA
500	Unweight	14	247	254	96		5	247	255	96
	Prentice-Wilcoxon	8	188	214	96		7	205	215	95
	$W_{n2}(t)$	-1	172	198	94		-3	188	200	94
	Shen et al. (2017)	-21	187	NA	NA		9	235	NA	NA
600	Unweight	11	222	229	96		7	227	232	96
	Prentice-Wilcoxon	5	173	195	96		5	186	196	95
	$W_{n2}(t)$	-4	155	180	95		-4	172	182	95
	Shen et al. (2017)	-20	164	NA	NA		-5	180	NA	NA

*SSE* The sampling standard deviation, *SEE* The sampling standard error, *Cov* The empirical coverage of approximate 95% confidence intervals

**Table 2:** Simulation results when ignoring truncation

		${\hat{\beta }}_n^{(1)}$					${\hat{\beta }}_n^{(2)}$			
n	Truncation	Bias$\times 10^3$	SSE$\times 10^3$	SEE$\times 10^3$	Cov(%)		Bias$\times 10^3$	SSE$\times 10^3$	SEE$\times 10^3$	Cov(%)
300	Mild	-39	284	309	95		-35	267	310	99
300	Moderate	-134	307	305	86		-74	297	308	96
300	Heavy	-306	555	629	91		-106	555	601	98
400	Mild	-67	229	260	95		-46	225	263	98
400	Moderate	-128	254	261	89		-71	265	264	96
400	Heavy	-379	245	251	70		-157	236	261	93
500	Mild	-66	200	229	96		-26	222	235	96
500	Moderate	-125	215	230	91		-72	227	234	96
500	Heavy	-398	215	222	55		-170	224	231	89
600	Mild	-53	173	209	96		-20	206	214	98
600	Moderate	-129	184	208	93		-72	213	212	93
600	Heavy	-406	223	202	45		-165	202	210	87

SSE, the sampling standard deviation; SEE, the sampling standard error; Cov, the empirical coverage of approximate 95% confidence intervals

To better illustrate how to employ the proposed method in real situation, we analyze the Centers for Disease Control’s blood-transfusion data, this data was used by Kalbfleisch and Lawless (1989) and Wang (1989). The data include 494 cases reported to the Center of Disease Control prior January, 1, 1987, and diagnosed before July, 1, 1986. Only 295 of the 494 has consistent data, and they got infection by a single blood transfusion or a short series of transfusions, analyse is restricted to this subset. We obtain the raw observation data via personal communication, Thomas Peterman, Centers for Disease Control and Prevention. The data contains three variables: *T* is the time from blood transfusion to the diagnosis of AIDS (in months), *R* is the time from blood transfusion to the end of the study (July, 1986, in months), Age is the age of the person when transfusing blood (in years). Comparing the data with Kalbfleisch and Lawless (1989)’s as well as Wang (1989)’s, the observation (*X*=16, *T*=33, Age=34) cannot be found in the raw data, thus is being deleted and the final sample size is 294, and a few fractions of the data are also corrected because these entries are not correct compared to the raw data.

We apply the proposed method to this data and treat Age as the covariate in regression. In Wang (1989)’s paper, the data are categorized into three age groups: ‘children’ aged 1-4, ‘adults’ aged 5-59, and ‘elderly patients’ aged 60 and older because of different patterns of survivorship, the survivor behaviour of groups ‘adults’ and ‘elderly patients’ are similar except for the right tail while there is an evident distinction compared with ‘children’, in current analysis, we delete the data from ‘children’, and focus on a combined sample of ‘adults’ and ‘elderly patients’ with a sample size equal to 260. Finally, the range of *T* is from 0 to 89, and the range of *R* is from 0 to 99. For all $i\in \{1,\dots ,260\}$, we have $T_i\le R_i$. As a result, our dataset will not have the identifiability issue as mentioned in Seaman et al. (2022). We also applied Shen et al. (2017)’s method and the result is similar. All the results are shown in Table , where the weights are chosen as $W_{n1}(t)$ and $W_{n2}(t)$, the estimated parameter between unweighted and weighted estimation equation does not show much difference, but the variance is reduced when weights are considered. In both situations mentioned above, Age has a very weakening positive effect on the odds ratio, but the effect is not significant.

**Table 3:** Age effect for blood transfusion data

	Age	SSE
unweighted	-0.0128	0.0153
Prentice-Wilcoxon	-0.0120	0.0143
$W_{n2}(t)$	-0.0122	0.0122
Shen et al. (2017)	-0.0125	0.0150

## Discussion

Directly consider the right truncated data in normal time order can be failed because ‘at risk’ process is not adapt to the history of the process (Gross 1992). Retro hazard solves this problem which transform right truncated data to left truncated in reverse time (Woodroofe 1985). Statistical modelling is even more flexible by incorporating the nature structure of proportional odds model. The usual form of proportional odds model can also be utilized but the theoretical and computational burden for the estimator will be increased, employ (1) can substantially improve the situation.

## Appendix 1

Assumptions:

A1: $\beta _0\in {\mathbb {R}}^p$ is the interior point of a compact set ${\mathcal {B}}$.

A2: *Z* is a bounded process.

A3: $V(\beta _0)$ is non-negative.

A4: *f*(*t*) is continuous.

Assumption A1 is also used by Chen et al. (2012), A2 is a standard assumption to ensure martingale properties holds (Fleming and Harrington 1991), A3 is also a standard assumption to avoid theoretical discussion, it is also being used in Huang and Qin (2013), A4 is being used in prove the martingale representation of ${\hat{v}}_n(t,\beta _0)-v(t)$. Besides that, we also need an condition to ensure that the truncated distribution to be correctly identified, let $F(\cdot )$ and *G*(*t*) be the distribution function of *T* and *R*, define $(a_F,b_F)$ and $(a_G,b_G)$ be the support of $F(\cdot )$ and $G(\cdot )$ of *T* and *R* under the meaning that $a_W=\inf \{x:W(x)>0\}, b_W=\sup \{ x:W(x)<1\}$, where *W* is a distribution function. Under right truncation, actually, only conditional distribution $P(T\le x|T\le b_G)$ and $P(R\le x| R\ge a_F)$ can be estimated, thus we assume $a_F=a_G=0$, $b_R=\infty$, so that the conditional distribution will be the actual distribution of *T* and *R*, we also assume $P(T\le Y)=\alpha >0$ to ensure that there exist observations satisfy our condition, similar assumption and discussion also appeared in Woodroofe (1985); Wang (1989), and Sundaram (2009), among others.

### Proof of Theorem 1

To prove the Theorem 1, the first step is to derive the martingale representation of ${\hat{S}}_n(\beta _0)$. To do this, we need the martingale representation of ${\hat{v}}_n(t,\beta _0)-v_0(t)$. Notice that

16 $$\begin{aligned}{} & {} \sum _{i=1}^n\{\exp (Z_i^\top \beta _0)v_0(t)+1\}dN_i(t)+\sum _{i=1}^n Y_i(t)\frac{dv_0(t)}{v_0(t)}\nonumber \\{} & {} \quad =\sum _{i=1}^n\{\exp (Z_i^\top \beta _0)v_0(t)+1\}M_i(dt,\beta _0)\end{aligned}$$

17 $$\begin{aligned}{} & {} \sum _{i=1}^n\{\exp (Z_i^\top \beta _0){\hat{v}}_n(t,\beta _0)+1\}dN_i(t)+\sum _{i=1}^n Y_i(t)\frac{{\hat{v}}_n(dt,\beta _0)}{dt}\frac{1}{{\hat{v}}_n(t,\beta _0)}=0.\nonumber \\ \end{aligned}$$

Denote $w_0(t)=1/v_0(t)$ and ${\hat{w}}_n(t,\beta )=1/{\hat{v}}_n(t,\beta )$, then (17) and (16) becomes:

18 $$\begin{aligned}{} & {} \sum _{i=1}^n\{\exp (Z_i^\top \beta _0)+w_0(t)\}dN_i(t)-\sum _{i=1}^n Y_i(t)dw_0(t)\nonumber \\{} & {} \quad =\sum _{i=1}^n\{\exp (Z_i^\top \beta _0)+w_0(t)\}M_i(dt,\beta _0),\end{aligned}$$

19 $$\begin{aligned}{} & {} \sum _{i=1}^n\{\exp (Z_i^\top \beta _0)+{\hat{w}}_n(t,\beta _0)\}dN_i(t)-\sum _{i=1}^n Y_i(t){\hat{w}}_n(dt,\beta _0)=0. \end{aligned}$$

(19)-(18) and divide both side by $-\sum _{i=1}^nY_i(t)$:

$$\begin{aligned}{} & {} \frac{\partial \{{\hat{w}}_n(t,\beta _0)-w_0(t)\}}{\partial t}- p_n(t)dt\{{\hat{w}}_n(t,\beta _0)-w_0(t)\}\\{} & {} \quad =\frac{\sum _{i=1}^n\{\exp (Z_i^\top \beta _0)+w_0(t)\}M_i(dt,\beta _0)}{\sum _{i=1}^nY_i(t)}. \end{aligned}$$

Then

$$\begin{aligned}{} & {} {\hat{w}}_n(t,\beta _0)-w_0(t) =\frac{1}{P_n(t)}\sum _{i=1}^n\int _t^{\tau }P_n(s) \frac{\left\{ \exp (Z_i^\top \beta _0)+w_0(s)\right\} }{\sum _{j=1}^nY_j(s)}M_i(ds,\beta _0). \end{aligned}$$

In the interval $(0,\tau )$, since $0<v_0(t)<\infty$, by delta method,

20 $$\begin{aligned} {\hat{v}}_n(t,\beta _0)-v_0(t){} & {} =-\frac{1}{w_0^2(t)}\left\{ {\hat{w}}_n(t,\beta _0)-w_0(t)\right\} \nonumber \\{} & {} =-\frac{v_0^2(t)}{P_n(t)}\sum _{i=1}^n\int _t^{\tau }P_n(s) \frac{v_0(s)\exp (Z_i^\top \beta _0)+1}{\sum _{j=1}^nY_j(s)v_0(s)}M_i(ds,\beta _0).\nonumber \\ \end{aligned}$$

At the point 0, (20) holds without condition because ${\hat{v}}_n(0,\beta )=v_0(t)=0$. At the point $\tau$, if denote $0\times \infty =0$, then (20) also holds.

By using (20), for $S_n(\beta _0)$:

$$\begin{aligned} S_n(\beta _0){} & {} =\frac{1}{n}\sum _{i=1}^n\int _0^{\tau }\left\{ Z_i-{\bar{Z}}(t)\right\} \left\{ \exp (Z_i^\top \beta _0){\hat{v}}_n(t,\beta _0)+1\right\} dN_i(t)\\{} & {} =\frac{1}{n}\sum _{i=1}^n\int _0^{\tau }\left\{ Z_i-{\bar{Z}}(t)\right\} \left\{ \exp (Z_i^\top \beta _0){\hat{v}}_n(t,\beta _0)+1\right\} M_i(dt,\beta _0)\\{} & {} \quad -\frac{1}{n}\sum _{i=1}^n\int _0^{\tau }\left\{ Z_i-{\bar{Z}}(t)\right\} Y_i(t) \frac{\exp (Z_i^\top \beta _0){\hat{v}}_n(t,\beta _0)+1}{\exp (Z_i^\top \beta _0)v_0^2(t)+v_0(t)}dv_0(t)\\{} & {} =\text {I}+\text {II}. \end{aligned}$$

In the following, we will show that the second part can also be represented as a summation of integral with respect to martingale.

21 $$\begin{aligned} \text {II}= & {} -\frac{1}{n}\sum _{i=1}^n\int _0^{\tau } \{Z_i-{\bar{Z}}(t)\}Y_i(t) \left\{ \frac{\exp (Z_i^\top \beta _0){\hat{v}}_n(t,\beta _0)+1-\exp (Z_i^\top \beta _0)v_0(t)-1}{\exp (Z_i^\top \beta _0)v_0^2(t)+v_0(t)}\right. \nonumber \\{} & {} \quad \left. +\frac{1}{v_0(t)} \right\} dv_0(t)\nonumber \\= & {} -\frac{1}{n}\sum _{i=1}^n\int _0^{\tau } \{Z_i-{\bar{Z}}(t)\}Y_i(t) \frac{\exp (Z_i^\top \beta _0)}{\exp (Z_i^\top \beta _0)v_0^2(t)+v_0(t)}\left\{ {\hat{v}}_n(t,\beta _0)-v_0(t)\right\} dv_0(t).\nonumber \\ \end{aligned}$$

Substitute (20) into (21) and change the integration order, then

$$\begin{aligned} \text {II}{} & {} =\frac{1}{n}\sum _{j=1}^n \int _0^{\tau } \left\{ P_n(t)\frac{\exp (Z_j^\top \beta _0)v_0(t)+1}{\sum _{k=1}Y_k(t)v_0(t)}\right. \\{} & {} \quad \left. \sum _{i=1}^n \int _0^t\{Z_i-{\bar{Z}}(s)\}Y_i(s) \frac{\exp (Z_i^\top \beta _0)v_0(s)}{\exp (Z_i^\top \beta _0)v_0(s)+1} \frac{1}{P_n(s)}dv_0(s) \right\} M_j(dt,\beta _0). \end{aligned}$$

Denote

$$\begin{aligned} \xi _i(t,\beta _0)= & {} \left\{ Z_i-{\bar{Z}}(t)\right\} \left\{ \exp (Z_i^\top \beta _0){\hat{v}}_n(t,\beta _0)+1\right\} \\{} & {} +P_n(t)\frac{\exp (Z_i^\top \beta _0)v_0(t)+1}{\sum _{k=1}Y_k(t)v_0(t)}\\{} & {} \times \sum _{j=1}^n \int _0^t\{Z_j-{\bar{Z}}(s)\}Y_j(s) \frac{\exp (Z_j^\top \beta _0)v_0(s)}{\exp (Z_j^\top \beta _0)v_0(s)+1} \frac{1}{P_n(s)}dv_0(s). \end{aligned}$$

Then the martingale representation of $S_n(\beta _0)$ is

22 $$\begin{aligned} S_n(\beta _0)=\frac{1}{n}\sum _{i=1}^n\int _0^{\tau }\xi _i(t,\beta _0)M_i(dt,\beta _0). \end{aligned}$$

Through (22), it is obvious to prove that $S_n(\beta _0)$ converges to zero 0 in probability by the weak law of large numbers.

Let

$$\begin{aligned} \mu (t)=\lim _{n\rightarrow \infty }\frac{\sum _{i=1}^nY_i(t)Z_i}{\sum _{j=1}^nY_j(t)}, v(t,\beta )=\lim _{n\rightarrow \infty }{\hat{v}}_n(t,\beta ). \end{aligned}$$

Denote

$$\begin{aligned} s_n(\beta )=\frac{1}{n}\sum _{i=1}^n\int _0^{\tau }\{Z_i-\mu (t)\}\left\{ \exp (Z_i^\top \beta ){\hat{v}}_n(t,\beta )-\exp (Z_i^\top \beta _0){\hat{v}}_n(t,\beta _0) \right\} dN_i(t). \end{aligned}$$

The derivative of $S_n(\beta )$ and $s_n(\beta )$ are

$$\begin{aligned}{} & {} S_n^{\prime }(\beta )=\frac{1}{n}\sum _{i=1}^n\{Z_i-{\bar{Z}}(t)\} \left\{ \exp (Z_i^\top \beta )Z_i{\hat{v}}_n(t,\beta )+\exp (Z_i^\top \beta )\{\partial {\hat{v}}_n(t,\beta )/\partial \beta \} \right\} dN_i(t),\\{} & {} s_n^{\prime }(\beta )=\frac{1}{n}\sum _{i=1}^n\{Z_i-\mu (t)\} \left\{ \exp (Z_i^\top \beta )Z_i{\hat{v}}_n(t,\beta )+\exp (Z_i^\top \beta )\{\partial {\hat{v}}_n(t,\beta )/\partial \beta \} \right\} dN_i(t). \end{aligned}$$

Notice $s_n(\beta _0)=0$. Assume that there exists $\varepsilon >0$ such that (A5): $P\{\mid Z_i-\mu (t)\mid>\varepsilon ,i=1,2,\cdots ,n\}>0$, which means covariate can not be identical for all individuals. Together with the assumption (A6):

$$\begin{aligned} \mid E\left\{ \exp (Z^\top \beta _0)Z{\hat{v}}_n(t,\beta _0)\right\} +E\left[ \exp (Z^\top \beta _0)\{\partial {\hat{v}}_n(t,\beta )/\partial \beta \}\mid _{\beta =\beta _0} \right] \mid >0. \end{aligned}$$

we have $\mid \lim _ns_n^{\prime }(\beta _0)\mid >0$. Without loss of generality, let $\lim _ns_n^{\prime }(\beta _0)>0$, then there exist a neighborhood of $\beta _0$ such that $s_n(\beta )$ is strictly increasing. Further notice that $S_n(\beta )=s_n(\beta )+o_p(1)$, $S_n^{\prime }(\beta )=s_n^{\prime }(\beta )+o_p(1)$, then $S_n(\beta )$ is strictly increasing in a neighborhood of $\beta _0$, thus prove the consistency of ${\hat{\beta }}_n$.

By martingale central limit theorem, the variance of $S_n(\beta _0)$ is

$$\begin{aligned} V(\beta _0){} & {} =\lim _{n\rightarrow \infty }V_n =\lim _{n\rightarrow \infty }<n^{-1/2}S_n(\beta _0),n^{-1/2}S_n(\beta _0)>(\tau )\\{} & {} =\lim _{n\rightarrow \infty }\frac{1}{n}\int _0^{\tau }\sum _{i=1}^n\xi _i(t,\beta _0)^{\otimes 2}d \int _t^{\tau }-Y_i(s) \{\exp (Z_i^\top \beta _0)v^2(s)+v(s)\}^{-1}dv(s)\\{} & {} =\lim _{n\rightarrow \infty }\frac{1}{n}\int _0^{\tau }\sum _{i=1}^n\xi _i(t,\beta _0)^{\otimes 2} Y_i(t) \{\exp (Z_i^\top \beta _0)v^2(t)+v(t)\}^{-1}dv(t). \end{aligned}$$

Further using the delta method will complete the proof of Theorem 1.

$$\square $$

### Proof of Theorem 2

Since Theorem 1 and 2 are quite similar, in this part, we will omit the proof detail and only give the detailed expression of $V_w$.

$$\begin{aligned} V_w(\beta _0) =\lim _{n\rightarrow \infty }\frac{1}{n}\int _0^{\tau }\sum _{i=1}^n\xi _{i,w}(t,\beta _0)^{\otimes 2} Y_i(t) \{\exp (Z_i^\top \beta _0)v^2(t)+v(t)\}^{-1}dv(t). \end{aligned}$$

where

$$\begin{aligned} \xi _{i,w}(t,\beta _0)= & {} W_n(t)\left\{ Z_i-{\bar{Z}}(t)\right\} \left\{ \exp (Z_i^\top \beta _0){\hat{v}}_n(t,\beta _0)+1\right\} \\{} & {} +P_n(t)\frac{\exp (Z_i^\top \beta _0)v_0(t)+1}{\sum _{k=1}Y_k(t)v_0(t)}\\{} & {} \times \sum _{j=1}^n \int _0^tW_n(s)\{Z_j-{\bar{Z}}(s)\}Y_j(s) \frac{\exp (Z_j^\top \beta _0)v_0(s)}{\exp (Z_j^\top \beta _0)v_0(s)+1} P_n(s)^{-1}dv_0(s). \end{aligned}$$

$$\square $$
